# 

**Published:** 1897-04

**Authors:** 


### CONTENTS:

### SELECTIONS.

Article I.—The Orbicularis Oris and the Muscles of Expression. By W. C. Barrett, M. D., D. D. S., - - - - 529
" II.—Cataphoresis in Dental Practice. By Dr. E. Grosheintz. - - - - - - - - - 539
" III.—Empyema of the Antrum of Highmore. By Edward E. Gibbons, M. D. - - - - - - 541
" IV.—The Latest Discovery. - - - - - - 543
" V.—Shall We Ask Congress for a Special Law Regulating Medical and Dental Patents? - - - - - 545
" VI.—Cataphoretic Extirpation of Living Pulps. By Vincent M. Murr, D. D. S. - - - - 552
" VII.—Pyorrhœa Not Infectious. By Dr. B. F. Arringtion 553
" VIII.—Diphtheria and Scarlet Fever. By G. E. Daly, M, D. 555
" IX.—The Inheritance of Mutilations and other Inheritances. By S. M. Worthington, M. D. - - 559
" X.—The Mouth Mirror. - - - - - - 562

### COMMUNICATED.

Dentists in the U. S. Army. - - - - - - 565

Illinois State Dental Society. - - - - - - 568

### EDITORIAL, ETC.

University of Maryland, Dental Department. - - - - 568
The Baltimore College of Dental Surgery. - - - - 570

### OBITUARY.

Moses Atwood Hopkins, D. D. S., - - - - - - 572

### MONTHLY SUMMARY.

Nodule on Apex of Root. - - - - - - - - 575

Creosote. - - - - - - - - - - 576

